# Diagnosis of Kawasaki Disease Using a Minimal Whole-Blood Gene Expression Signature

**DOI:** 10.1001/jamapediatrics.2018.2293

**Published:** 2018-08-06

**Authors:** Victoria J. Wright, Jethro A. Herberg, Myrsini Kaforou, Chisato Shimizu, Hariklia Eleftherohorinou, Hannah Shailes, Anouk M. Barendregt, Stephanie Menikou, Stuart Gormley, Maurice Berk, Long Truong Hoang, Adriana H. Tremoulet, John T. Kanegaye, Lachlan J. M. Coin, Mary P. Glodé, Martin Hibberd, Taco W. Kuijpers, Clive J. Hoggart, Jane C. Burns, Michael Levin

**Affiliations:** 1 Section of Paediatrics, Imperial College London, London, United Kingdom; 2 Department of Pediatrics, University of California San Diego, La Jolla; 3 Rady Children’s Hospital–San Diego, San Diego, California; 4 Department of Pediatric Hematology, Immunology & Infectious Diseases, Emma Children’s Hospital, Academic Medical Centre, University of Amsterdam, Amsterdam, the Netherlands; 5 Infectious Diseases, Genome Institute of Singapore, Singapore; 6 Department of Genomics of Common Disease, School of Public Health, Imperial College London, London, United Kingdom; 7 Institute for Molecular Bioscience, The University of Queensland, St Lucia, Australia; 8 Section of Infectious Diseases, Department of Pediatrics, University of Colorado Denver School of Medicine Anschutz Medical Campus, Aurora; 9 Children’s Hospital Colorado, Aurora; 10 Sanquin Research and Landsteiner Laboratory, Department of Blood Cell Research, Academic Medical Centre, University of Amsterdam, Amsterdam, the Netherlands

## Abstract

**Importance:**

To date, there is no diagnostic test for Kawasaki disease (KD). Diagnosis is based on clinical features shared with other febrile conditions, frequently resulting in delayed or missed treatment and an increased risk of coronary artery aneurysms.

**Objective:**

To identify a whole-blood gene expression signature that distinguishes children with KD in the first week of illness from other febrile conditions.

**Design, Setting, and Participants:**

The case-control study comprised a discovery group that included a training and test set and a validation group of children with KD or comparator febrile illness. The setting was pediatric centers in the United Kingdom, Spain, the Netherlands, and the United States. The training and test discovery group comprised 404 children with infectious and inflammatory conditions (78 KD, 84 other inflammatory diseases, and 242 bacterial or viral infections) and 55 healthy controls. The independent validation group comprised 102 patients with KD, including 72 in the first 7 days of illness, and 130 febrile controls. The study dates were March 1, 2009, to November 14, 2013, and data analysis took place from January 1, 2015, to December 31, 2017.

**Main Outcomes and Measures:**

Whole-blood gene expression was evaluated using microarrays, and minimal transcript sets distinguishing KD were identified using a novel variable selection method (parallel regularized regression model search). The ability of transcript signatures (implemented as disease risk scores) to discriminate KD cases from controls was assessed by area under the curve (AUC), sensitivity, and specificity at the optimal cut point according to the Youden index.

**Results:**

Among 404 patients in the discovery set, there were 78 with KD (median age, 27 months; 55.1% male) and 326 febrile controls (median age, 37 months; 56.4% male). Among 202 patients in the validation set, there were 72 with KD (median age, 34 months; 62.5% male) and 130 febrile controls (median age, 17 months; 56.9% male). A 13-transcript signature identified in the discovery training set distinguished KD from other infectious and inflammatory conditions in the discovery test set, with AUC of 96.2% (95% CI, 92.5%-99.9%), sensitivity of 81.7% (95% CI, 60.0%-94.8%), and specificity of 92.1% (95% CI, 84.0%-97.0%). In the validation set, the signature distinguished KD from febrile controls, with AUC of 94.6% (95% CI, 91.3%-98.0%), sensitivity of 85.9% (95% CI, 76.8%-92.6%), and specificity of 89.1% (95% CI, 83.0%-93.7%). The signature was applied to clinically defined categories of definite, highly probable, and possible KD, resulting in AUCs of 98.1% (95% CI, 94.5%-100%), 96.3% (95% CI, 93.3%-99.4%), and 70.0% (95% CI, 53.4%-86.6%), respectively, mirroring certainty of clinical diagnosis.

**Conclusions and Relevance:**

In this study, a 13-transcript blood gene expression signature distinguished KD from other febrile conditions. Diagnostic accuracy increased with certainty of clinical diagnosis. A test incorporating the 13-transcript disease risk score may enable earlier diagnosis and treatment of KD and reduce inappropriate treatment in those with other diagnoses.

## Key Points

**Question:**

Can Kawasaki disease be accurately diagnosed on the basis of the pattern of host gene expression in whole blood?

**Findings:**

In this case-control study of 606 children (404 in the discovery cohort; 202 in the validation cohort), a 13-transcript signature was identified that accurately discriminated Kawasaki disease from comparator febrile diseases in discovery and validation cohorts.

**Meaning:**

A diagnostic blood test based on measurement of small numbers of host gene transcripts might enable early discrimination of Kawasaki disease from other infectious and inflammatory conditions.

## Teaser

This case-control study identifies a whole-blood gene expression signature that distinguishes children with Kawasaki disease in the first week of illness from other febrile conditions.

## Introduction

Kawasaki disease (KD) is an acute inflammatory disorder predominantly seen in young children. Since it was first described in Japan,^1^ KD has emerged as the most common cause of acquired heart disease, with an incidence in children younger than 5 years ranging from 265 cases per 100 000 in Japan,^2^ to 51 to 194 cases per 100 000 in other Asian countries,^3,4,5^ to 8 to 20 cases per 100 000 in Europe^6^ and the United States,^7^ respectively. What makes KD of such concern is its association with vasculitis, affecting predominantly the coronary arteries, which results in coronary artery aneurysms (CAAs) in up to 25% of untreated children.^8^ Death from myocardial infarction may occur due to thrombotic occlusion of the aneurysms or from the later development of stenotic lesions due to vascular remodeling in the damaged artery. Long-term outcome studies^9,10^ of children with giant CAAs indicate a worrisome prognosis, with more than 50% needing revascularization or experiencing myocardial infarction within a 30-year period.

Treatment with intravenous immunoglobulin (IVIG) and, for those who do not respond, additional IVIG^11^ or other anti-inflammatory agents, such as corticosteroids and infliximab, is effective in abrogating the inflammatory process and reduces the risk of CAAs to 5% to 10%.^12^ Because KD is difficult to distinguish from other common febrile conditions, many children with KD are not diagnosed and treated early enough to prevent development of CAAs.^13^ Furthermore, patients who do not fulfill the clinical criteria for diagnosing KD (so-called incomplete KD) may experience CAAs. Delayed diagnosis is a consistent risk factor for development of CAAs, and treatment is often commenced only when coronary dilatation is already demonstrated on echocardiography.

The symptoms of KD are similar to those of several other childhood febrile illnesses, including staphylococcal and streptococcal toxic shock syndromes, measles and other viral illnesses (eg, adenovirus infection, Rocky Mountain spotted fever), and childhood inflammatory diseases, leading to diagnostic difficulty and thus delay in diagnosis and treatment. Guidelines have been developed to facilitate diagnosis based on clinical signs and symptoms, echocardiography, and laboratory variables,^14^ but there remains an urgent need for an accurate test to distinguish KD from other conditions causing prolonged fever in children.

In the era of precision medicine, diagnosis of many conditions previously based on clinical features alone is being replaced by diagnosis based on molecular pathology. Host blood gene expression signatures have been shown to identify several specific infectious and inflammatory diseases, including tuberculosis,^15^ bacterial and viral infections,^16,17^ and systemic lupus erythematosus.^18^ Support for a diagnostic approach to KD based on gene expression signatures comes from identification of microRNA biomarkers in KD,^19,20^ although existing studies are limited by the range of comparator patients or a need to extract RNA from exosomes. We explored use of whole-blood gene expression patterns to distinguish KD from other childhood infectious and inflammatory conditions. We present a gene expression signature, discovered and validated in independent patient groups, that distinguishes KD from a range of bacterial, viral, and inflammatory illnesses.

## Methods

### Ethical Approval and Informed Consent

Patients were recruited, with written parental informed consent, under approvals by the research ethics committees of the United Kingdom (St Mary’s Hospital 09/H0712/58, 13/LO/0026); Spain (Ethical Committee of Clinical Investigation of Galicia [CEIC] 2010/015); Amsterdam, the Netherlands (NL41023.018.12 and NL34230.018.10); and the University of California San Diego (Human Research Protection Program 140220).

### Patient Study Groups

The differential diagnosis for KD includes multiple infectious and inflammatory conditions. Therefore, in this case-control study, we established a discovery group of children with KD and a range of other infectious and inflammatory diseases with clinical signs, inflammatory markers, and duration of fever overlapping KD. Patients were prospectively recruited at pediatric centers in the United Kingdom, Spain, the Netherlands, and the United States if they had febrile illness and required blood testing for clinical investigation as part of the UK-based Immunopathology of Respiratory, Inflammatory and Infectious Disease Study (IRIS)^17^; the Spanish GENDRES (Genetic, Vitamin D, and Respiratory Infections Research Network) study (http://www.gendres.org); the Dutch Kawasaki Study; or the US-based Kawasaki Disease Research Center Program (https://medschool.ucsd.edu/som/pediatrics/research/centers/kawasaki-disease). The training and test discovery group comprised 404 children with infectious and inflammatory conditions (78 KD, 84 other inflammatory diseases, and 242 bacterial or viral infections) and 55 healthy controls. The independent validation group comprised 102 patients with KD, including 72 in the first 7 days of illness, and 130 febrile controls. The study dates were March 1, 2009, to November 14, 2013, and data analysis took place from January 1, 2015, to December 31, 2017.

Children with KD represented a combination of those seen directly in emergency departments and patients referred from regional centers. Our study included only patients recruited before initiation of IVIG for treatment. For discovery of a diagnostic signature, we included patients with KD in the first 7 days of illness because we aimed to develop a test for use early in the illness before coronary artery damage occurs. However, to explore the performance of the signature at all stages of illness, we included patients up to day 10 of their illness in the validation study.

**Figure 1.**
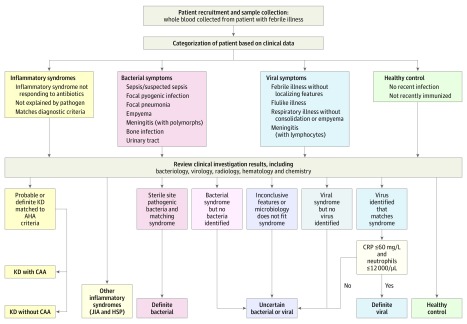
Assignment of Patients to Diagnostic Groups The diagnostic algorithm demonstrates the method of assigning patients to diagnostic groups. AHA indicates American Heart Association; CAA, coronary artery aneurysm; CRP, C-reactive protein; HSP, Henoch-Schönlein purpura; JIA, juvenile idiopathic arthritis; and KD, Kawasaki disease. To convert C-reactive protein level to nanomoles per liter, multiply by 9.524; to convert neutrophil count to ×10^9^/L, multiply by 0.001.

Febrile controls whose duration of illness before hospital presentation varied were recruited, with blood samples collected as soon as possible after presentation and before clinical diagnosis was confirmed. Febrile controls were assigned to diagnostic groups using predefined criteria once the results of all investigations were available (Figure 1 and eMethods in the Supplement). Children with comorbidities likely to influence gene expression, such as immunosuppressive treatments, were excluded. We included comparator groups of children seen with inflammatory illness, including juvenile idiopathic arthritis and Henoch-Schönlein purpura. Comparison of the duration of illness, inflammatory markers. and demographics between patients with KD and febrile controls is summarized in Table 1.

**Table 1.** Clinical Characteristics and Initial Laboratory Values for Patients With Kawasaki Disease and Febrile Controls in Discovery and Validation Study Groups^a^

Variable	Discovery Set		Validation Set	
	Kawasaki Disease	Febrile Controls^b^	Kawasaki Disease^c^	Febrile Controls^b^
No. of patients	78	326	72	130
Age, median (IQR), mo	27 (16 to 45)	37 (9 to 116)	34 (17 to 51)	17 (5 to 47)
Male sex, No. (%)	43 (55.1)	184 (56.4)	45 (62.5)	74 (56.9)
Illness day at sample collection, median (IQR)^d^	5 (4 to 6)	6 (4 to 9)	5 (5 to 6)	5 (3 to 7)
Laboratory values, median (IQR)				
Hemoglobin *z* score^e^	−1.3 (−2.0 to −0.3)	NA	−1.2 (−2.0 to −0.4)	NA
C-reactive protein, mg/L	119 (48 to 192)	66 (23 to 174)	87 (59 to 173)	62 (16 to 162)
Platelet count, ×10^3^/μL	352 (303 to 448)	254 (167 to 351)	408 (324 to 474)	277 (176 to 352)
White blood cell count, /μL	14 200 (10 400 to 18 300)	8000 (6000 to 12 900)	13 900 (11 000 to 19 000)	11 000 (7700 to 16 000)
Neutrophil count, /μL	9000 (6600 to 12400)	5000 (3100 to 9400)	10000 (7300 to 12600)	7000 (3600 to 13400)
Ethnicity, No. (%)				
No. not stated^f^	0	23 (7.1)	0	10 (8.3)
African	3 (3.8)	28 (8.6)	2 (2.8)	23 (19.2)
Asian, including Indian subcontinent and Far East	12 (15.4)	29 (8.9)	12 (16.7)	12 (10.0)
European	20 (25.6)	186 (57.1)	20 (27.8)	68 (56.7)
Hispanic	25 (32.1)	20 (6.1)	14 (19.4)	0
Mixed	15 (19.2)	28 (8.6)	23 (31.9)	7 (5.8)
Other	3 (3.8)	12 (3.7)	1 (1.4)	10 (8.3)
Coronary artery status, No. (%)				
Normal	45 (57.7)	NA	52 (72.2)	NA
Dilated	25 (32.1)	NA	15 (20.8)	NA
Aneurysm	8 (10.3)	NA	5 (6.9)	NA
IVIG resistant, No. (%)	18 (23.1)	NA	15 (20.8)	NA

Abbreviations: IQR, interquartile range; IVIG, intravenous immunoglobulin; NA, not applicable.

SI conversation factors: To convert C-reactive protein level to nanomoles per liter, multiply by 9.524; neutrophil count to ×10^9^/L, multiply by 0.001; platelet count to ×10^9^/L, multiply by 1.0; and white blood cell count to ×10^9^/L, multiply by 0.001.

^a^ There were no significant differences between patients with Kawasaki disease in the discovery and validation sets.

^b^ Healthy controls were not included.

^c^ Data refer to the 72 patients in the first week of Kawasaki disease.

^d^ Illness day 1 is the first day of fever (in Kawasaki disease) or symptoms (in febrile controls).

^e^ Hemoglobin was normalized by age (data unavailable for febrile controls).

^f^ Ethnicity percentages were calculated for the denominator with recorded data.

Patients in the validation study group were similarly recruited as part of biomarker studies of febrile children seen in the hospital and requiring blood tests, as described previously.^21,22^ Healthy control children with no recent history of fever or immunization were recruited alongside patients with KD and febrile control patients in the discovery and validation studies. Data from healthy controls were used to standardize data obtained in different microarray experiments but were not used to evaluate the performance of the signature.

### KD Case Definition

Kawasaki disease was diagnosed on the basis of the American Heart Association criteria,^14^ with 2-dimensional echocardiography performed soon after presentation (2 and 6 weeks after onset). Patients with fewer than 4 of the 5 classic criteria (bilateral nonpurulent conjunctivitis, oral mucosal changes, peripheral extremity changes, rash, and cervical lymphadenopathy >1.5 cm) were included as having incomplete KD if the maximum coronary artery *z* score (Zmax) (standard deviation units from the mean internal diameter normalized for body surface area) at any time during the illness for the left anterior descending or right coronary arteries was 2.5 or higher or if the patients satisfied the algorithm for incomplete KD in the American Heart Association guidelines. Patients were classified as having normal (Zmax <2.5), small (Zmax 2.5 to <5.0), or large (Zmax ≥5.0) CAAs. Because of interoperator variability in coronary artery dimensions, we set a high (Zmax ≥5.0) threshold to define patients with confirmed aneurysms and thus definite diagnosis of KD.

### Further Classification of KD by Diagnostic Certainty

Because there is no gold standard for diagnosis, some patients may meet the criteria for KD but have other conditions. Therefore, we further categorized patients with KD in the validation study group based on certainty of clinical diagnosis. All clinical records, laboratory results, echocardiogram reports, response to treatment, and follow-up were reviewed by an independent pediatric infectious disease specialist and expert on KD (M.P.G.) masked to the analysis. Patients with documented CAAs (Zmax ≥5.0) persisting 6 weeks after onset were considered to have definite KD because there is no other known self-resolving inflammatory illness in childhood that leads to CAAs. The remaining patients (all of whom were treated with IVIG for suspected KD) were classified as having highly probable, possible, or unlikely KD by the expert reviewer. This review identified no unlikely KD cases.

### Febrile Control Children With Infection or Other Inflammatory Syndromes

Children seen with febrile illnesses were recorded as having definite bacterial infection, definite viral infection, suspected bacterial or viral infection, juvenile idiopathic arthritis, or Henoch-Schönlein purpura. The criteria shown in Figure 1 and described in eMethods in the Supplement were used to make this determination.

### Oversight and Conduct of the Study

Patients were categorized into disease groups (Figure 1) after evaluation of all results by 2 independent clinicians not involved in the patients’ care (J.A.H., A.M.B., J.T.K., M.P.G., and J.C.B.). All blood samples were anonymized, and transcriptomic data sets were analyzed only after clinical assignments were finalized and dispatched for independent verification (eMethods in the Supplement).

### Discovery and Validation of the Gene Expression Signature

**Figure 2.**
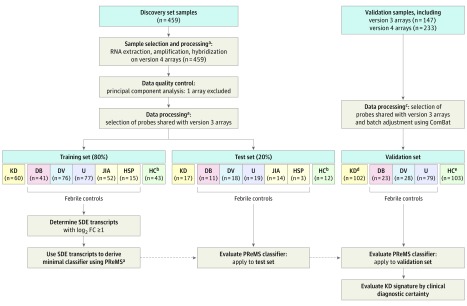
Study Design The overall study pipeline shows sample handling, derivation of test and training data sets, data processing, and analysis pipeline. Version 3 arrays indicate HumanHT-12, version 3.0 BeadChip (Illumina); version 4 arrays indicate HumanHT-12, version 4.0 BeadChip (Illumina); and ComBat indicates the ComBat algorithm.^23^ DB indicates definite bacterial; DV, definite viral; FC, fold change; HC, healthy controls; HSP, Henoch-Schönlein purpura; JIA, juvenile idiopathic arthritis; KD, Kawasaki disease; PReMS, parallel regularized regression model search; SDE, significantly differentially expressed; and U, infections of uncertain bacterial or viral etiology. ^a^See Supplemental Methods (RNA sample extraction and processing), as well as Statistical Methods in eMethods in the Supplement. ^b^Healthy controls were used in model building but were excluded from estimates of model accuracy. ^c^See Statistical Methods in eMethods in the Supplement; 146 acute KD samples (HumanHT-12, version 4.0) were used in Combat, of which 101 were taken forward. ^d^Diagnostic performance was assessed on 72 patients (within the first 7 days of illness). ^e^Includes convalescent KD and healthy controls.

The overall study design and signature discovery pipeline are shown in Figure 2. Whole blood was collected at the time of recruitment into blood RNA tubes (PAXgene; PreAnalytiX), frozen, extracted, and analyzed on arrays (HumanHT-12 version 4.0 BeadChip; Illumina). An earlier array (HumanHT-12 version 3.0 BeadChip; Illumina) with largely overlapping probes was used in a subset of the validation study group. Details of laboratory methods are provided in eMethods in the Supplement.

### Statistical Analysis

#### Transcript Signature Discovery

Analysis of the transcriptomic data was conducted with statistical software (R, version 3.2.2; R Foundation for Statistical Computing). As shown in Figure 2, the discovery study group was randomly divided into an 80% training set and a 20% test set. The signature was identified in the training set and validated in the test set and in the validation study group, established using previously reported acute and convalescent patients with KD^21^ and acute bacterial and viral patients^22^ (eMethods in the Supplement). After quality control and filtering (eMethods in the Supplement), significantly differentially expressed transcripts in patients with KD compared with all other diseases were identified in the training set.

#### Small Signature Discovery Using Parallel Regularized Regression Model Search

A range of statistical methods are available to identify signatures from significantly differentially expressed transcripts, including least absolute shrinkage and selection operator (LASSO)^24^ and elastic net.^25^ However, these approaches produce large signatures that may not be easy to translate into a bedside diagnostic test. Therefore, we developed a novel variable selection method, parallel regularized regression model search, that identifies and ranks transcript signatures on the basis of their least number of transcripts and highest accuracy in discrimination^26^ (eMethods in the Supplement). The method first evaluates all possible 1- and 2-transcript models distinguishing KD from comparator diseases based on all significantly differentially expressed transcripts and takes the 100 best-fitting 2-transcript models to the next round, when a further transcript is added to the model and all combinations are again evaluated. The process continues with the incremental addition of 1 further transcript at a time to the best 100 models. The optimal signature for a given number of transcripts (model size) was selected after ranking each model by its Watanabe-Akaike information criterion, which is a Bayesian estimate of the out-of-sample error.^27^ The optimal model size was determined by cross-validation.

#### Disease Risk Score and Assessment of Model Accuracy

We applied the previously reported disease risk score (DRS) method that assigns individual disease risk based on the transcripts in the diagnostic signature.^15^ The DRS combines the fluorescence intensity of upregulated transcripts and subtracts the combined fluorescence intensity of down-regulated transcripts^15^ and might facilitate development of tests from complex signatures. Healthy controls were used in model building but were excluded from estimates of model accuracy, assessed by area under the curve (AUC), sensitivity, and specificity at the optimal cut point according to the Youden index.

## Results

The numbers of patients in each diagnostic category are shown in Figure 2. Clinical and demographic features of patients with KD and febrile controls are summarized in Table 1, with further details of control patients listed in eTable 1 in the Supplement. Principal component analysis of the normalized transcript expression profiles was performed separately on the discovery (training and test) and validation groups (eFigure 1 and eFigure 2 in the Supplement). Study groups clustered together in the discovery group and in the validation group after combining KD and case-control data using the ComBat algorithm^23^ (Statistical Methods in eMethods in the Supplement).

### Identification of Minimal Transcript Signatures

**Figure 3.**
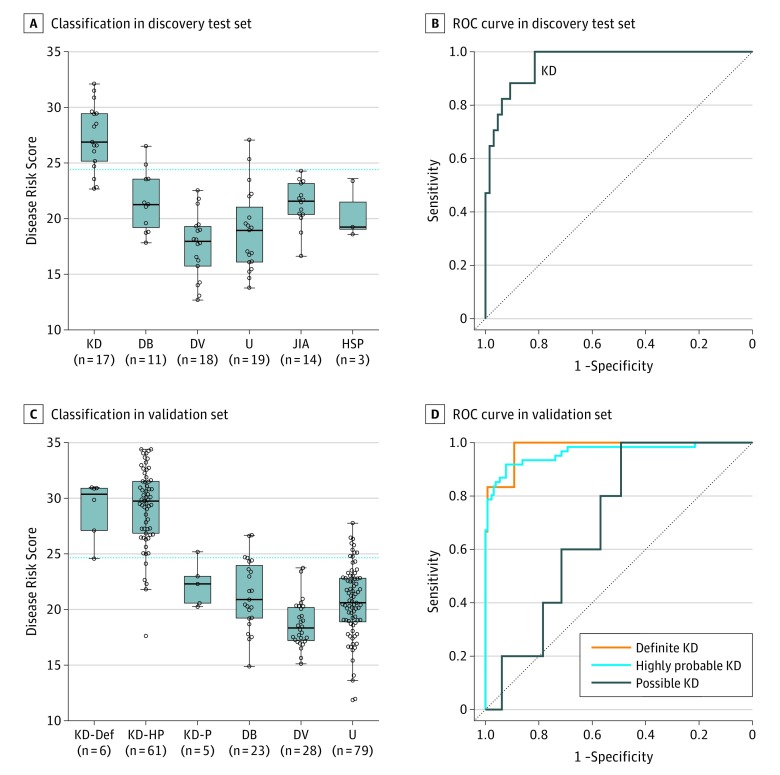
Performance of the 13-Transcript Signature on the Discovery Test Set and the Validation Set Shown is classification (A) and ROC curve (B) of the 13-transcript signature in the discovery test set, comprising patients with KD and patients with other diseases, using the disease risk score. Shown is classification (C) and ROC curves (D) of the 13-transcript signature in the validation set, comprising 3 KD clinical subgroups of differing diagnostic certainty and patients with other diseases. In box plots, horizontal lines represent the median; lower and upper edges represent interquartile ranges; and whiskers represent the range or 1.5 times the interquartile range, whichever is smaller. The horizontal blue line indicates the disease risk score threshold that separates patients predicted as having KD (above the line) or not having KD (below the line) as determined by the point in the ROC curve that maximized sensitivity and specificity in the discovery training group. DB indicates definite bacterial; DV, definite viral; HSP, Henoch-Schönlein purpura; JIA, juvenile idiopathic arthritis; KD, Kawasaki disease; KD-Def, definite KD; KD-HP, highly probable KD; KD-P, possible KD; ROC, receiver operating characteristic; and U, infections of uncertain bacterial or viral etiology.

In total, 1600 transcripts passed quality control and were significantly differentially expressed between KD and all other diseases and healthy controls (defined as log_2_ fold change >1 in KD vs at least 1 of the comparator groups). To identify small signatures suitable for developing as a diagnostic test, we next undertook variable selection using parallel regularized regression model search. This approach identified a 13-transcript signature (Table 2) that, when implemented as a DRS, had a diagnostic performance in the discovery test set distinguishing KD from other infectious and inflammatory conditions, with an AUC of 96.2% (95% CI, 92.5%-99.9%), sensitivity of 81.7% (95% CI, 60.0%-94.8%), and specificity of 92.1% (95% CI, 84.0%-97.0%) (Figure 3A and B).

**Table 2.** Genes Included in the Diagnostic Signature

Gene Symbol	Gene Name	HGNC Identification No.	Probe Identification No.	Location	Logistic Regression Coefficient^a^
*CACNA1E*	Calcium voltage-gated channel subunit alpha1 E	1392	7510647	1q25.3	0.955
*DDIAS*	DNA damage–induced apoptosis suppressor	26351	2570019	11q14.1	0.844
*KLHL2*	Kelch-like family member 2	6353	1070593	4q32.3	0.789
*PYROXD2*	Pyridine nucleotide-disulphide oxidoreductase domain 2	23517	1684497	10q24.2	0.727
*SMOX*	Spermine oxidase	15862	270068	20p13	0.675
*ZNF185*	Zinc finger protein 185 with domain	12976	6840674	Xq28	0.646
*LINC02035*	Long intergenic non–protein coding RNA 2035	52875	3236239	3q21.1	0.561
*CLIC3*	Chloride intracellular channel 3	2064	5870136	9q34.3	0.464
*S100P*	S100 calcium-binding protein P	10504	1510424	4p16.1	−0.405
*IFI27*	Interferon alpha–inducible protein 27	5397	3990170	14q32.12	−0.426
*HS.553068*	BX103476 NCI_CGAP_Lu5 *Homo sapiens* cDNA clone	NA	1470450	NA	−0.599
*CD163*	CD163 molecule	1631	2680092	12p13.31	−0.638
*RTN1*	Reticulon 1	10467	6860193	14q23.1	−0.690

Abbreviations: cDNA, complementary DNA; HGNC, Hugo Gene Nomenclature Committee; NA, not applicable.

^a^ The logistic regression coefficient indicates the power of the gene to discriminate Kawasaki disease in the parallel regularized regression model search. Genes with positive values show increased expression in Kawasaki disease relative to other diseases, and genes with negative values show decreased expression in Kawasaki disease.

### Signature Performance in Validation Set

When the signature was applied to all of the 72 KD cases in the validation set, who were in the first 7 days of illness, the AUC was 94.6% (95% CI, 91.3%-98.0%), with sensitivity of 85.9% (95% CI, 76.8%-92.6%) and specificity of 89.1% (95% CI, 83.0%-93.7%). The performance was slightly reduced in the 30 patients diagnosed later (days 8-10) (eTable 2 and eFigure 3 in the Supplement).

Because clinical features of KD overlap those of other conditions and because any KD study group is likely to include patients misclassified as KD, we assessed whether certainty of clinical diagnosis corresponded to the predictive performance of the KD DRS. The performance of the 13-transcript signature in the patients with definite, highly probable, or possible KD in the validation set mirrored certainty of clinical diagnosis, with AUCs of 98.1% (95% CI, 94.5%-100%), 96.3% (95% CI, 93.3%-99.4%), and 70.0% (95% CI, 53.4%-86.6%), respectively (Figure 3C and D and eTable 2 in the Supplement).

## Discussion

We identified a 13-transcript signature that distinguishes patients with KD from patients with bacterial, viral, and inflammatory illnesses. The high sensitivity and specificity of this signature for early diagnosis of KD suggests that it might form the basis of a diagnostic test. Our findings herein extend previous gene expression studies^21,28,29,30,31,32^ in KD that focused on immunopathogenesis.

The diagnosis of KD now relies on the presence of 4 of the 5 characteristic clinical criteria. Fewer criteria are accepted if coronary artery abnormalities (dilatation or aneurysms) are detected on echocardiography. Children with incomplete KD who do not fulfil the classic diagnostic criteria but have prolonged fever and inflammation are at an increased risk of developing CAAs.^33^ One reason for the greater risk of CAAs in incomplete KD is the delayed diagnosis that often occurs in patients lacking all clinical features. Because the clinical features of KD overlap those of many other common childhood conditions,^34^ treatment with IVIG may be delayed while awaiting exclusion of other conditions. Conversely, because the diagnosis of KD is considered in the differential diagnosis of many childhood febrile illnesses and because the consequences of delayed treatment may be severe, overtreatment with IVIG or immunosuppressant second-line treatments may occur. A diagnostic test that accurately distinguishes KD from other infectious and inflammatory processes would be a significant advance in management of the disorder, reduce unnecessary investigations and inappropriate treatments, and enable earlier treatment with IVIG and other anti-inflammatory agents.

In establishing our discovery and validation study groups, we aimed to include a wide range of disorders with features overlapping those of KD, including both infectious and inflammatory diseases. The signature that we have identified distinguished KD from a wide range of other conditions with similar duration of fever and overlapping levels of inflammation. Because KD is diagnosed based on a constellation of clinical features and because there is no gold standard for diagnosis, evaluation of biomarker test results is difficult. Any cohort of children treated with IVIG for presumed KD is likely to include some patients with non-KD illness but with similar features. To evaluate the correspondence of the KD DRS with levels of diagnostic certainty, we categorized patients in the validation set as having definite, highly probable, or possible KD based on independent review of the clinical data. We observed a higher sensitivity and specificity of our signature in the definite and highly probable KD groups than in the possible KD group.

Regarding the transcripts in the signature (Table 2), expression was lower in patients with KD compared with the non-KD group for 5 of the 13 transcripts. Of these, *S100P*, previously reported to have increased expression in acute KD relative to convalescence^35^ or viral infections,^32,35^ was most abundant in patients with bacterial infection. The *IFI27* gene has been reported to be upregulated in children with viral compared with bacterial infections^36^ and autoimmune diseases,^37,38^ consistent with reduced expression of genes induced by type 1 interferons reported in acute KD vs adenovirus infection.^32^ CD163 is a transmembrane receptor expressed in macrophages and monocytes involved in bacterial clearance during acute infection.^39^ A network analysis of the signature using pathway analysis (Ingenuity Pathways Analysis; Ingenuity Systems) revealed that 7 of the 13 transcripts in the signature were connected in a network around a central hub of tumor necrosis factor and interleukin 6 (eFigure 4 in the Supplement).

### Strengths and Limitations

We recognize both strengths and limitations in our study. First, the epidemiology of KD varies globally by ethnicity. Although we included patients with a mix of ethnicities in both discovery and validation cohorts, further studies are required to establish the performance of the signature in other geographic populations. Second, in the validation experiment, data from different Illumina microarray versions and studies were combined using the ComBat algorithm to achieve normalization. This normalization may reduce both experimental and biological sources of variability between data sets; consequently, the accuracy (AUC) of the signature in the validation set may be an underestimate. Conversely, although we showed that the ComBat algorithm successfully normalized the data sets, residual batch associations may have falsely increased the performance of the signature. Third, to develop a signature applicable in a wide range of febrile conditions, we discovered the signature through comparison of KD cases with a wide range of febrile controls with a spectrum of KD diagnosis likelihoods. This potentially biased the test toward better discriminatory value than might be applicable in the clinical setting. Fourth, we discovered the signature using patients with KD in the first 7 days of illness, with the aim of identifying a test for use early in the disease to enable treatment before coronary injury has occurred. Because the performance of the signature was lower in patients with KD seen after the seventh day, further work is required to establish the optimal signature for diagnosis in patients with KD with late presentation.

## Conclusions

The results of our study suggest that KD can be distinguished from the range of infectious and inflammatory conditions with which it is often clinically confused using 13 transcripts in blood. Development of a test based on this gene expression signature is made more achievable because of the small number of transcripts in our signature and the rapidly evolving technologies for detecting nucleic acids. A diagnostic test would be a major advance allowing earlier treatment and thus prevention of cardiac complications of this serious childhood disease. Our findings represent a step toward better diagnosis of diseases based on molecular signatures rather than the clinical criteria and are relevant to many other clinical syndromes.

## Notes

Supplement. **eMethods.** Supplemental Methods **eTable 1A.** Clinical Features of Children in the Juvenile Idiopathic Arthritis Cohort (Discovery) **eTable 1B.** Clinical Features of Children in the Henoch-Schönlein Purpura Group (Discovery) **eTable 1C.** Clinical Features of Children With Bacterial and Viral Infection, Infections of Uncertain Bacterial or Viral Etiology, and Healthy Controls (Discovery and Validation) **eTable 1D.** Viral and Bacterial Causative Pathogens in Patients in the Definite Bacterial and Viral Groups **eTable 2.** Summary of Performance of Models **eFigure 1.** Principal Component Analysis on the Discovery Cohort **eFigure 2.** Principal Component Analysis on Validation Sets Before and After Merging Using ComBat **eFigure 3.** Performance of the 13-Transcript Signature by Illness Day at Sample Collection in Validation Set **eFigure 4.** Gene Network Derived From 13-Transcript Signature
