# Supplementary material for: Diagnosis of Kawasaki Disease Using a Minimal Whole-Blood Gene Expression Signature
Source: JAMA Pediatr. 2018 Aug 6;172(10):e182293. doi: 10.1001/jamapediatrics.2018.2293 (PMC6233768; doi:10.1001/jamapediatrics.2018.2293)
Supplement: Supplement. — eMethods. Supplemental Methods eTable 1A. Clinical Features of Children in the Juvenile Idiopathic Arthritis Cohort (Discovery) eTable 1B. Clinical Features of Children in the Henoch-Schönlein Purpura Group (Discovery) eTable 1C. Clinical Features of Children With Bacterial and Viral Infection, Infections of Uncertain Bacterial or Viral Etiology, and Healthy Controls (Discovery and Validation) eTable 1D. Viral and Bacterial Causative Pathogens in Patients in the Definite Bacterial and Viral Groups eTable 2. Summary of Performance of Models eFigure 1. Principal Component Analysis on the Discovery Cohort eFigure 2. Principal Component Analysis on Validation Sets Before and After Merging Using ComBat eFigure 3. Performance of the 13-Transcript Signature by Illness Day at Sample Collection in Validation Set eFigure 4. Gene Network Derived From 13-Transcript Signature [file jamapediatr-172-e182293-s001.pdf]

Supplementary Online Content

Wright VJ, Herberg JA, Kaforou M, et al; Immunopathology of Respiratory, Inflammatory and Infectious Disease Study (IRIS) Consortium and the Pediatric Emergency Medicine Kawasaki Disease Research Group (PEMKDRG). Diagnosis of Kawasaki disease using a minimal whole-blood gene expression signature. *JAMA Pediatr*. Published online August 6, 2018. doi:10.1001/jamapediatrics.2018.2293

eMethods. Supplemental Methods
eTable 1A. Clinical Features of Children in the Juvenile Idiopathic Arthritis Cohort (Discovery)
eTable 1B. Clinical Features of Children in the Henoch-Schönlein Purpura Group (Discovery)
eTable 1C. Clinical features of children with bacterial and viral infection, infections of uncertain bacterial or viral aetiology and healthy controls (Discovery and Validation)
eTable 1D. Viral and Bacterial causative pathogens in patients in the Definite Bacterial and Viral groups
eTable 2. Summary of Performance of Models
eFigure 1. Principal Component Analysis on the Discovery Cohort
eFigure 2. Principal Component Analysis on Validation Sets Before and After Merging Using ComBat
eFigure 3. Performance of the 13-Transcript Signature by Illness Day at Sample Collection in Validation Set
eFigure 4. Gene Network Derived From 13-Transcript Signature
eReferences

This supplementary material has been provided by the authors to give readers additional information about their work.

© Wright VJ. *JAMA Pediatrics.*

eMethods. Supplemental Methods

RNA sample extraction and processing

Whole blood (2.5ml) was collected into PAXgene blood RNA tubes (PreAnalytiX, Germany), incubated for 2 hours, frozen at -20ºC within 6 hours of collection, before storage at -80ºC. RNA was extracted using PAXgene blood RNA kits (PreAnalytiX, Germany) according to the manufacturer's instructions. The integrity and yield of the total RNA was assessed using an Agilent 2100 Bioanalyser and a NanoDrop 1000 spectrophotometer. The samples used in the discovery cohort came from the USA (UCSD), Spain, The Netherlands and UK. All samples were extracted in the UK except for the samples from the USA. After quantification and quality control, biotin-labeled cRNA was prepared using Illumina TotalPrep RNA Amplification kits (Applied Biosystems) from 500ng RNA. Labeled cRNA was hybridized overnight to Human HT-12 v.4 Expression BeadChip arrays (Illumina). After washing, blocking and staining, the arrays were scanned using an Illumina BeadArray Reader according to the manufacturer's instructions. Using Genome Studio software the microarray images were inspected for artifacts and QC parameters were assessed. No arrays were excluded at this stage.

Pathogen diagnosis

Viral diagnostics were undertaken on nasopharyngeal aspirates using immunofluorescence (RSV, adenovirus, parainfluenza virus, influenza A+B) and nested PCR (RSV, adenovirus, parainfluenza 1-4, influenza A+B, bocavirus, metapneumovirus, rhinovirus/enterovirus). Bacterial cultures included blood, CSF, urine and tissue sites. Pneumococcal antigen was measured in blood and urine, and bacterial DNA was detected by meningococcal and pneumococcal PCR.

Diagnostic process in febrile controls

Patients had a diagnostic work-up as directed by the clinical team, including blood count, blood chemistry, C-reactive protein (CRP), blood urine and throat swab cultures; cerebrospinal fluid analysis and chest radiographs were performed where appropriate. Multiplex PCR was used to detect common respiratory viruses in nasopharyngeal aspirates or throat swabs, and common viruses in blood. Once the results of all investigations were available, patients were assigned to diagnostic groups using predefined criteria (Figure 1), as follows:

Bacterial infection: Patients assigned to the bacterial pathogen group had a bacterial pathogen (gram-positive coccus or gram-negative bacillus) identified by culture or by molecular techniques in a sample from a sterile site (blood, CSF, pleural space, joint, urine), and a clinical syndrome in keeping with the identified bacterial species. This group included patients with and without viral co-infection. Children diagnosed with other bacterial infections (for instance mycoplasma, pertussis, mycobacteria) were not included in this group. No threshold for inflammatory markers was set for this group, as identification of bacteria in a sterile-site sample was taken as conclusive evidence for a confirmed bacterial infection.

Viral infection: Patients in the viral infection group had an identified virus, a clinical syndrome in keeping with viral infection, and no microbiological or clinical features of bacterial disease. In order to avoid inclusion of children with occult bacterial infection in the viral group, children with raised inflammatory markers were excluded. A maximum threshold was set at CRP of 60mg/L, and neutrophil count of 12 x 10^9/L. Among the 94 children, the most frequent pathogens were RSV (27 children), influenza A/B and adenovirus (23 children each).

Uncertain bacterial or viral infection: When children with an acute febrile illness and features of infection could not be assigned confidently to one of the above groups, they were labelled as 'Uncertain Bacterial or Viral'. Children in this group had inconclusive features of bacterial or viral infection, negative microbiological findings or absent virological investigations, a syndrome inconsistent with their microbiological findings, inflammatory markers inconsistent with other clinical features of their illness, or insufficient clinical data for confident coding in another group. Patients in this group did not have bacterial infection detected at a sterile site, and some patients did have detectable virus.

Other inflammatory syndromes: a) Henoch–Schönlein purpura **(HSP)** was diagnosed in children presenting with palpable purpura, typically over the buttocks and extensor surfaces in association with abdominal pain, arthralgia or renal abnormalities (hematuria and proteinuria); b) Juvenile idiopathic arthritis **(JIA)** was defined according to International League of Associations for Rheumatology[1]. Patients with JIA included i) treatment-naïve and ii) active-exacerbation/smouldering.

© Wright VJ. *JAMA Pediatrics.*

Statistical Methods

Microarray pre-processing - The Discovery Dataset

Background subtraction and robust spline normalisation (RSN) were applied to the raw expression data using the R package lumi[2]. Sample outliers were assessed by Principal Component Analysis (PCA). One sample from a Kawasaki patient, was a clear outlier on PC1 and was removed from the analysis (eFigure 1).

The samples in the discovery dataset were randomly assigned to ten different folds conditional on equal numbers of each comparator group (KD Kawasaki Disease, DB Definite Bacterial, DV Definite Viral, U infections of uncertain bacterial or viral aetiology, JIA juvenile idiopathic arthritis, HSP Henoch–Schönlein purpura, HC healthy controls). Two folds (20%) were reserved as the test set and the remaining eight folds made up the training set. As a diagnostic test for KD would be of most value early in the course of the illness, we developed our signature using only samples from patients at 7 or fewer days of fever in the discovery cohort.

Microarray pre-processing - The Validation Dataset

The validation dataset was constructed by merging two gene-expression datasets: one with acute and convalescent Kawasaki samples[3] and one with bacterial and viral infections[4]. All convalescent samples had ESR (erythrocyte sedimentation rate) levels less than 40mm/hr and all acute samples were taken within ten days of onset of illness. Background subtraction and RSN normalisation were applied to the two datasets separately in the R package lumi[2]. At this stage, there were differences between the cohorts. This is evident from a PCA plot which shows that PC1 clearly distinguishes samples by batch (eFig. 2a). We therefore employed the ComBat[5] method to remove batch effects. Two binary covariates were passed to ComBat which assigned samples to three groups - healthy, KD and other diseases. The Kawasaki convalescent samples were assigned as healthy. A PCA after ComBat shows samples from both batches overlap on a plot of PC1 against PC2 with no significant batch effects (eFig. 2b). T-tests for difference in Principal Component values between healthy controls and convalescent KD patients were non-significant (PC1 $p=0.32$; PC2 $p=0.98$).

Model estimation

Before model estimation probes were pre-filtered to identify robustly expressed transcripts with $\log_2$ fold change ≥ 1 between the relevant disease groups. This was implemented by selecting probes meeting all of the following criteria in the training data:

1. Probes measured on both V3 and V4 Illumina Beadchips
2. Robustly expressed transcripts: for each probe, we calculated the proportion of samples in each comparator group for which the detection threshold p-value<0.01, and selected those probes for which this proportion was > 80% in at least one disease group
3. The majority of Kawasaki patients were recruited in UCSD. To ensure probe selection was not biased by batch effects emanating from UCSD, we excluded probes which showed association with recruitment at UCSD at $p<0.05$ in a linear model conditional on age in months and all disease groups which also included non-KD patients recruited from UCSD (DV, U, KD and HSP)
4. $\log_2$ fold change (conditional on age) was calculated between Kawasaki and each other comparator group; we took forward those probes with $|\log_2 \text{fold change}|>1$ for at least one of these comparisons

The functions lmFit and eBayes in the R package limma[6] were used to calculate probe association statistic used in steps (3) and (4) above.

Discovery using Parallel Regularised Regression Model Search (PReMS)

We used PReMS, to derive a parsimonious gene-expression signature, which balances small transcript number with accurate discrimination[7]. The PReMS method used the pre-filtered transcripts that were robustly expressed with $\log_2$ fold change ≥ 1 between groups. PReMS uses cross-validation and selects the optimal model size as the one which minimises the out-of-sample log-likelihood. In the analysis presented in this paper, 20 cross-validation folds were used.

Calculating model accuracy

The area under ROC curves, and corresponding confidence intervals of the models' application to the test and validation datasets were calculated using the R package pROC[8].

Results for each patient were summarised as a Disease Risk Score (DRS) to determine the accuracy of classification by the 13-transcript signature, and the optimal threshold-cut-off for classification as KD or not KD, based on training set data, was determined according to Youden's J statistic by the point in the ROC curve

© Wright VJ. *JAMA Pediatrics.*

that maximizes the distance to the identity line (maximum of (sensitivities + specificities))[9]. The same threshold was used in accuracy calculations for the validation data.

Confidence intervals (CI) for sensitivity and specificity were calculated using Jeffrey's method. Jeffrey's method is derived from a Bayesian perspective in which the underlying proportion of interest is assigned the non-informative Jeffrey's reference prior: Beta(½, ½)[10]. Thus, sensitivity 95% CIs are derived from the 2.5% and 97.5% quantiles of a Beta (p+½, q+½) distribution, where p is the number of true positives and q is the number of false negatives.

© Wright VJ. *JAMA Pediatrics.*

eTable 1A. Clinical Features of Children in the Juvenile Idiopathic Arthritis Cohort (Discovery)

	Treatment-naive	Active-exacerbation/ smouldering
**No. children**	30	36
**Age, months[a]**	163.5 (124.0 – 186.8)	157 (137.8 – 176.5)
**Male, n (%)**	11 (37)	14 (39)
**Ethnicity, n (%)**		
**Caucasian**	27 (90)	26 (72)
**Turkish**	1 (3)	1 (2)
**Arabic**		4 (11)
**Black**	1 (3)	2 (5)
**Indian**		1 (2)
**Mixed**	1 (3)	2 (5)
**White blood count (x 10^3/mm^3)[a, b]**	6.3 (5.2 – 6.9)	5.9 (5.1 - 6.8)
**% neutrophils**	50.6 (44.9 - 57.4)	54.9 (46.1 - 60.2)
**% lymphocytes**	37.0 (32.9 - 44.7)	34.1 (29.4 - 41.0)
**% monocytes**	7.0 (6.3 – 8.0)	7.1 (5.8 - 7.8)
**% eosinophils**	2.3 (1.6 - 4.6)	2.7 (1.3 – 4.0)
**% basophils**	0.4 (0.3 - 0.8)	0.4 (0.2 - 0.5)
**ESR (mm/hour)[a]**	5 (2 - 10.5)	5 (2 - 9)
**C-reactive protein (mg/L)[a]**	0.9 (0.0 – 2.1)	0.9 (0.3 – 3.1)
**ANA positive (%)**	8 (26)	17 (47)
**ANCA positive (%)**	0	0

[a]All values shown as median (IQR); [b]Lab values out of 27 patients for treatment-naïve set, 35 patients for active-exacerbation/smouldering set. ESR = erythrocyte sedimentation rate, ANA = antinuclear antibodies, ANCA = anti-neutrophil cytoplasmic antibodies

© Wright VJ. *JAMA Pediatrics.*

eTable 1B. Clinical Features of Children in the Henoch-Schönlein Purpura Group (Discovery)

	Henoch-Schönlein Purpura
**No. children**	18
**Age, months[a]**	55.5 (43.0 – 81.0)
**Male, n (%)**	9 (50)
**Ethnicity, n (%)**	
**Caucasian**	4 (22)
**Hispanic**	4 (22)
**Mixed**	8 (44)
**Other**	2 (11)
**Illness day at sample collection[a]**	3.5 (2 - 6)
**White blood count (x 10^3/mm^3)[a]**	12.7 (9.7 – 14.2)[c]
**% neutrophils**	60.0 (45.0 – 67.5)[c]
**% bands**	3.5 (0.0 – 9.8)[d]
**% lymphocytes**	26.0 (15.8 – 33.9)[c]
**% monocytes**	7.0 (4.6 – 8.1)[c]
**% eosinophils**	1.0 (0.0 – 2.1)[c]
**Hemoglobin z-score[a,b]**	-0.1 (-0.7 – 0.6)[d]
**Platelet count (x 10^3/mm^3)[a]**	356.0 (321.0 – 488.5)[d]
**ESR (mm/hour)[a]**	23 (11 – 35.3)[e]
**C-reactive protein (mg/L)[a]**	22 (8 - 24)[f]

[a]All values shown as median (IQR); [b]Hemoglobin normalized by age; [c]Lab data available from 15 patients; [d]Lab data available from 14 patients; [e]Lab data available from 4 HSP; [f]Lab data available from 8 patients; ESR = erythrocyte sedimentation rate.

© Wright VJ. *JAMA Pediatrics.*

eTable 1c: Clinical features of children with bacterial and viral infection, infections of uncertain bacterial or viral aetiology and healthy controls (Discovery and Validation)

	Discovery group				Validation group			
	Definite bacterial	Definite viral	Uncertain	Healthy control	Definite bacterial	Definite viral	Uncertain	Healthy control
**No. children**	52	94	96	55	23	28	79	16
**Age, months[a]**	22 (9-46)	14 (2-39)	27 (7-71)	38 (20-77)	22 (13-52)	18 (7-48)	15 (2-44)	65 (44, 65)
**Male, n (%)**	22 (42)	66 (70)	62 (65)	29 (53)	10 (43%)	17 (61%)	47 (59%)	10 (63)
**Ethnicity, n (%)[b]**								
**Asian**	5 (10)	5 (6)	18 (21)	5 (10)	2 (9)	2 (7)	8 (11)	2 (13)
**Black**	2 (4)	11 (13)	12 (14)	5 (10)	5 (23)	4 (14)	14 (20)	1 (6)
**Caucasian**	35 (73)	47 (53)	47 (55)	21 (44)	12 (55)	14 (52)	42 (59)	8 (50)
**Hispanic**	0 (0)	14 (16)	2 (2)	0 (0)	0 (0)	0 (0)	0 (0)	0 (0)
**Middle East**	2 (4)	1 (1)	0 (0)	4 (8)	0 (0)	3 (11)	2 (3)	3 (19)
**Others**	4 (8)	10 (11)	6 (7)	13 (27)	3 (14)	4 (15)	5 (7)	2 (13)
**Not stated**	4	6	11	7	1	1	8	0
**Symptom days[a,c]**	5 (2-8.8)	4.5 (3.0-6.0)	5 (4.8-8)	n/a	4 (2.5-8)	3.5 (2.8-5.3)	4 (3-7)	n/a
**Intensive care, n (%)**	36 (69%)	34 (36%)	57 (59%)	n/a	13 (57)	7 (23)	42 (53)	n/a
**Deaths, n**	10	0	2	n/a	1	1	8	n/a
**White blood count (x 10^3/mm^3) [a]**	12.7 (7.7-19.3)	8.5 (6.1-12.0)	8.4 (6.5-14.6)	7.2 (6.4-9.75)	16.6 (10.0-19.3)	8.3 (5.6-10.9)	10.6 (6.5-16.0)	8.0 (5.8-8.9)
**% neutrophil**	75 (49-85)	50 (36-64)	63 (46-79)	45 (35-50)	82 (71-88)	53 (41-69)	64 (43-82)	45 (37-49)
**% lymphocyte**	19 (10-36)	34 (19-44)	22 (15-42)	44 (39-56)	15 (8-23)	32 (26-48)	30 (14-42)	43 (38-50)
**% monocyte**	5 (3-8)	10 (4-14)	6 (2-12)	6.5 (5.3-7.1)	3 (0-7)	7 (5-10)	5 (2-8)	7 (6-8)
**% eosinophil**	0 (0-1.2)	0 (0-1.0)	0 (0-0.9)	2.8 (1.6-5.6)	0 (0-0.4)	0 (0-2)	0 (0-1)	2.8 (2-5)
**C-reactive protein[a,d] (mg/L)**	176 (98-275)	14 (6-27)	102 (47-176)	n/a	217 (168-285)	7 (1-20)	67 (25-128)	n/a

[a]All values shown as median (IQR); [b]percentage of those with known ethnicity, [c]until research blood sampling, [d]maximum value of CRP in illness is reported.

© Wright VJ. *JAMA Pediatrics.*

eTable 1d: Viral and Bacterial causative pathogens in patients in the Definite Bacterial and Viral groups

	Definite Viral		Definite Bacterial	
	**Discovery**	**Validation**	**Discovery**	**Validation**
**Viral causative pathogen**				
**Adenovirus**	23	2		
**Influenza A or B**	23	13		
**RSV**	27	10		
**Other**	21	3		
**Bacterial causative pathogen**				
S.pneumoniae			10	15
S.aureus			2	2
S.pyogenes			10	10
**Group B streptococcus**			4	
E.coli			2	
N.meningitidis			17	
**Enterococcus**			1	
Kingella			1	
H.influenzae			1	
***Pseudomonas* spp**			3	
Stenotrophomonas			1	
Klebsiella				1
**Total**	**94**	**28**	**52**	**23**

© Wright VJ. *JAMA Pediatrics.*

eTable 2. Summary of Performance of Models

	Discovery group		Validation group			Validation group (n=72) separated by likelihood of KD		
	**Training set**	**Test set**	**KD patients ≤ day 7 (n=72)**	**KD patients > day 7 (n=30)**	**All KD patients (n=102)**	**Definite KD**	**Highly probable KD**	**Possible KD**
AUC	0.990	0.962	0.946	0.925	0.940	0.981	0.963	0.700
95% CI	0.982, 0.998	0.925, 0.999	0.913, 0.980	0.869, 0.981	0.910, 0.970	0.945, 1.000	0.933, 0.994	0.534, 0.866
Sensitivity	0.980	0.817	0.859	0.797	0.842			
95% CI	0.925, 0.998	0.600, 0.948	0.768, 0.926	0.633, 0.912	0.763, 0.904			
Specificity	0.930	0.921	0.891	0.899	0.899			
95% CI	0.898, 0.955	0.840, 0.970	0.830, 0.937	0.840, 0.943	0.840, 0.943			
KD positive, test positive	59	14	62					
KD positive, test negative	1	3	10					
Not KD, test positive	20	4	13					
Not KD, test negative	284	61	117					

© Wright VJ. *JAMA Pediatrics.*

eFigure 1. Principal Component Analysis on the Discovery Cohort

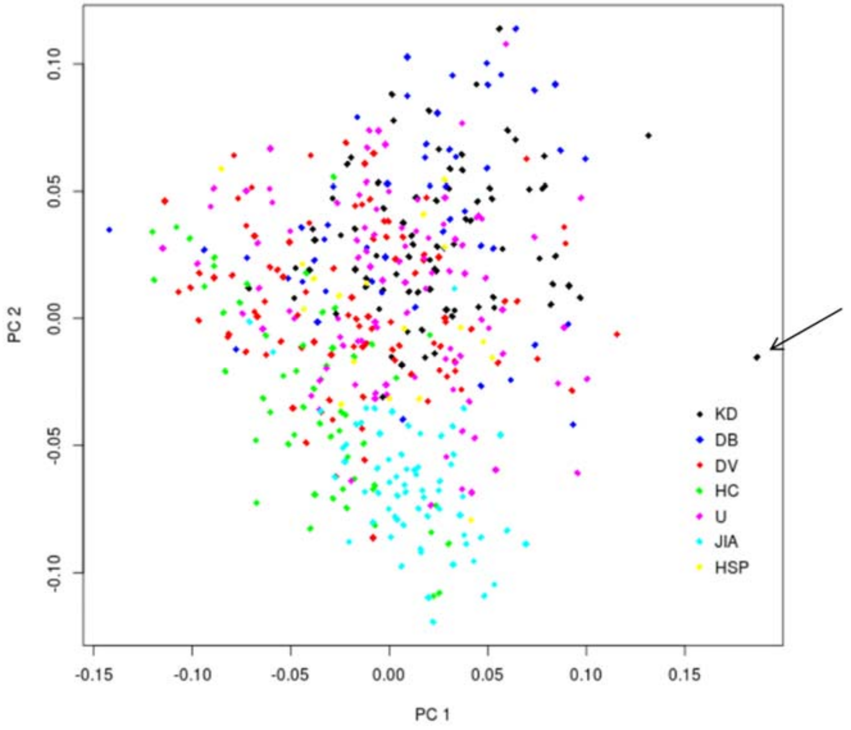

PCA plot of PC1 & PC2 in the discovery cohort after background adjustment and normalisation. A sample from a KD patient was removed (arrow) from subsequent analysis. Each spot is data from an array. KD Kawasaki Disease, DB Definite Bacterial, DV Definite Viral, HC healthy controls, U infections of uncertain bacterial or viral aetiology, JIA juvenile idiopathic arthritis, HSP Henoch-Schönlein purpura.

© Wright VJ. *JAMA Pediatrics.*

eFigure 2. Principal Component Analysis on Validation Sets Before and After Merging Using ComBat

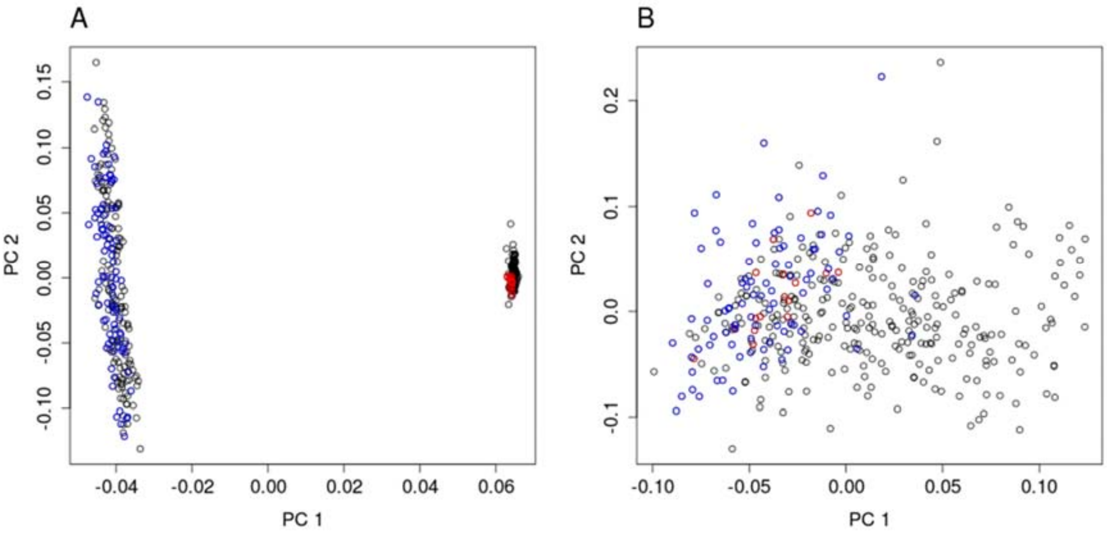

PCA plots of validation cohorts (a) pre-ComBat and (b) merging post-ComBat. Each spot represents data from an array; red circles represent healthy controls, blue circles represent convalescent KD; black circles represent acute KD and other diseases included in the febrile controls. Panel (b) includes data from 30 KD patients with samples after the 7th day of fever, who were not included in the diagnostic performance calculations. T-tests for difference in Principal Component values between healthy controls and convalescent KD patients were non-significant (PC1 p=0.32; PC2 p=0.98).

© Wright VJ. *JAMA Pediatrics.*

eFigure 3. Performance of the 13-Transcript Signature by Illness Day at Sample Collection in Validation Set

A

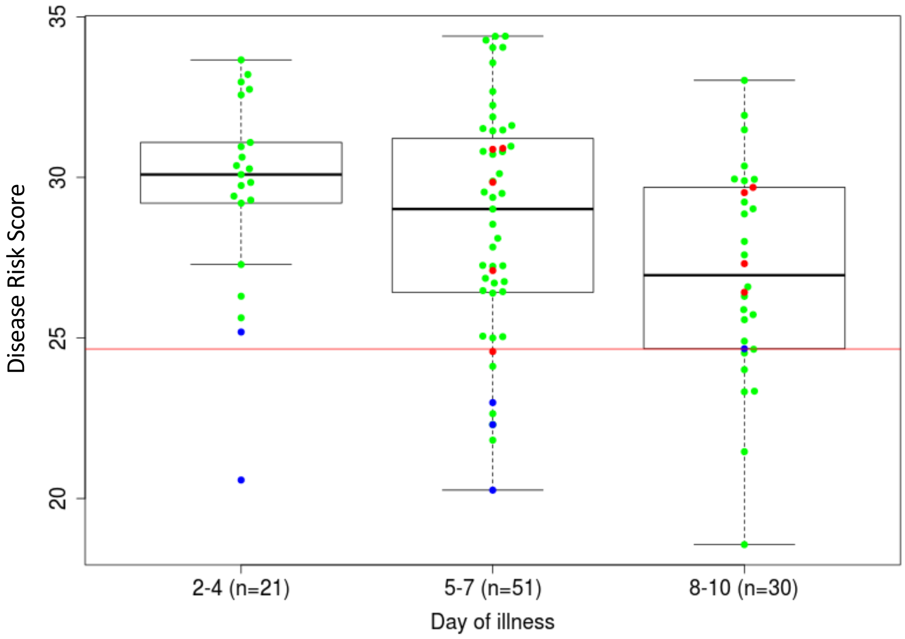

B

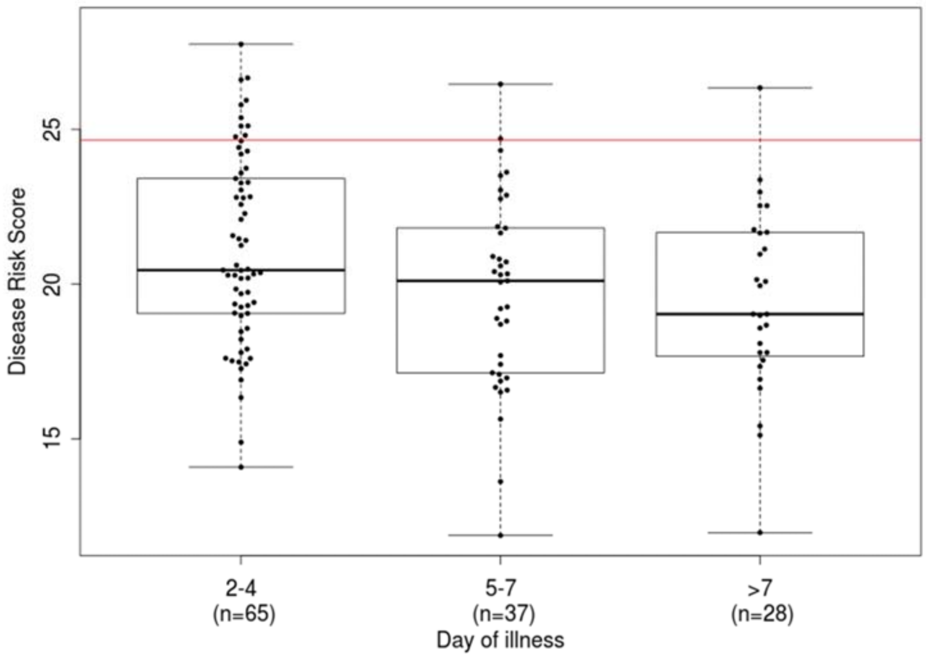

Variation in DRS according to day of illness in (A) the validation set KD patients, and in (B) the febrile controls. The X axis shows the collection day of the sample in relation to the first day of illness (i.e. initiation of fever). In panel A, colours of red, green and blue dots correspond, respectively, to the definite, highly probable and possible Kawasaki Disease clinical subgroups in the validation set.

© Wright VJ. *JAMA Pediatrics.*

eFigure 4. Gene Network Derived From 13-Transcript Signature

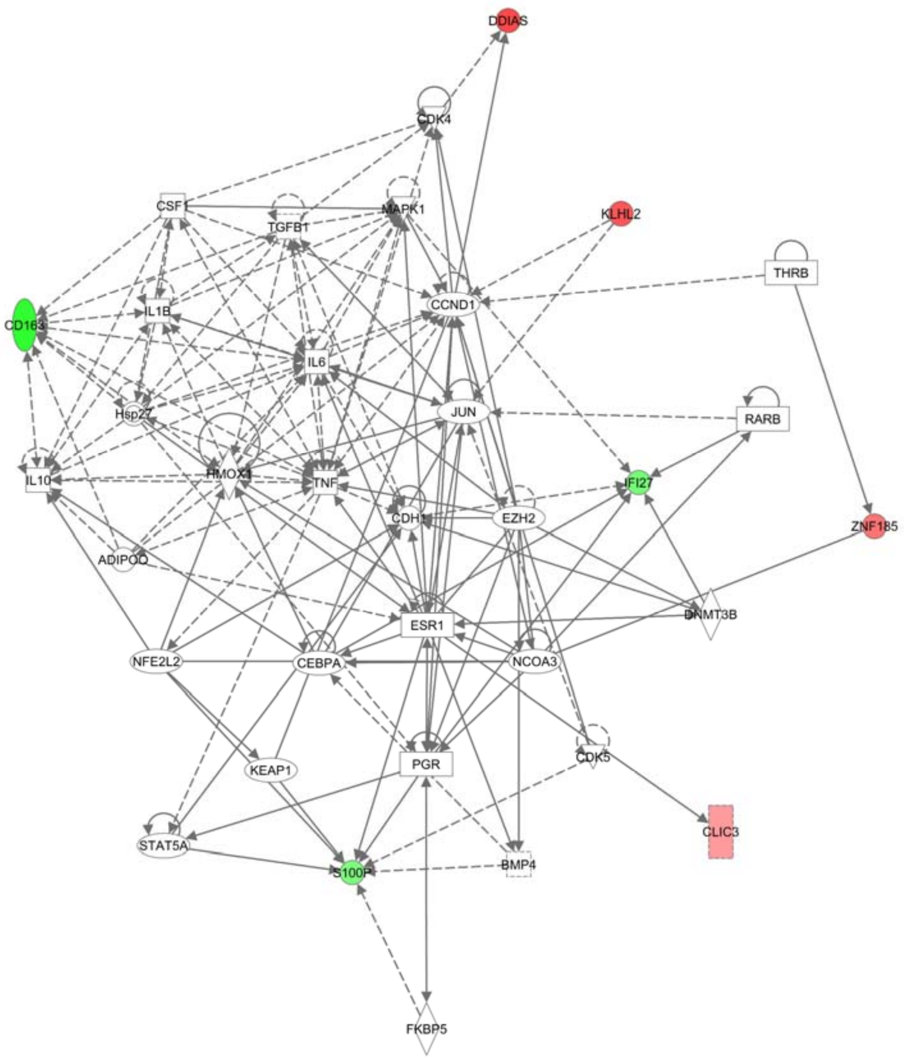

The network was generated using Ingenuity Pathways Analysis. 12 of the 13 transcripts were mapped to the database. This network, containing 7 focus molecules was the top network in the analysis. Each molecule is coloured according to the direction of expression in KD. Unbroken lines indicate direct interaction, dashed lines indicate indirect interaction.

The legend to the network is located at http://ingenuity.force.com/ipa/articles/Feature_Description/Legend.

© Wright VJ. *JAMA Pediatrics.*

References

1. Petty RE, Southwood TR, Manners P, et al. International League of Associations for Rheumatology classification of juvenile idiopathic arthritis: second revision, Edmonton, 2001. *J Rheumatol.* 2004;31(2):390-392.
2. Du P, Kibbe WA, Lin SM. lumi: a pipeline for processing Illumina microarray. *Bioinformatics.* 2008;24(13):1547-1548.
3. Hoang LT, Shimizu C, Ling L, et al. Global gene expression profiling identifies new therapeutic targets in acute Kawasaki disease. *Genome Med.* 2014;6(11):541.
4. Herberg JA, Kaforou M, Gormley S, et al. Transcriptomic profiling in childhood H1N1/09 influenza reveals reduced expression of protein synthesis genes. *The Journal of infectious diseases.* 2013;208(10):1664-1668.
5. Johnson WE, Li C, Rabinovic A. Adjusting batch effects in microarray expression data using empirical Bayes methods. *Biostatistics.* 2007;8(1):118-127.
6. Ritchie ME, Phipson B, Wu D, et al. limma powers differential expression analyses for RNA-sequencing and microarray studies. *Nucleic Acids Res.* 2015;43(7):e47.
7. Hoggart CJ. PReMS: Parallel Regularised Regression Model Search for sparse bio-signature discovery. *bioRxiv.* 2018.
8. Robin X, Turck N, Hainard A, et al. pROC: an open-source package for R and S+ to analyze and compare ROC curves. *BMC bioinformatics.* 2011;12:77.
9. Youden WJ. Index for rating diagnostic tests. *Cancer.* 1950;3(1):32-35.
10. Bernardo JM, Smith AFM. *Bayesian theory.* Chichester, Eng.; New York: Wiley; 1994.

© Wright VJ. *JAMA Pediatrics.*
